# In vitro anti-nephrotoxic potential of *Ammi visnaga*, *Petroselinum crispum*, *Hordeum vulgare*, and *Cymbopogon schoenanthus* seed or leaf extracts by suppressing the necrotic mediators, oxidative stress and inflammation

**DOI:** 10.1186/s12906-019-2559-8

**Published:** 2019-06-25

**Authors:** Marwa M. Abu-Serie, Noha H. Habashy, Adham M. Maher

**Affiliations:** 1 Department of Medical Biotechnology, Genetic Engineering, and Biotechnology Research Institute, City of Scientific Research and Technological Applications (SRTA-City), New Borg EL-Arab, Alexandria, 21934 Egypt; 2 Biochemistry Department, Faculty of Science, Alexandria University, Alexandria, 21511 Egypt

**Keywords:** Anti-nephrotoxicity, Necrosis, Oxidative stress, Inflammation, Plant extract

## Abstract

**Background:**

The kidney is an essential organ required by the body to perform several important functions. Nephrotoxicity is one of the most prevailing kidney complications that result from exposure to an extrinsic or intrinsic toxicant, which increase the need for the acquisition of proper remedies. Recently, natural remedies are gaining great attention owed to the fact that they have fewer side effects than most conventional drugs.

**Methods:**

The current study recorded a new therapeutic role of the well-known medicinal plants for kidney stones [*Ammi visnaga* (AVE), *Petroselinum crispum* (PCE), *Hordeum vulgare* (HVE), and *Cymbopogon schoenanthus* (CSE)]. Hence, the aqueous extracts of these plants examined against CCl_4_-induced toxicity in mammalian kidney (Vero) cells.

**Results:**

These extracts showed the presence of varying amounts of phenolic and triterpenoid compounds, as well as vitamin C. Owing to the antioxidant potential of these constituents, the extracts suppressed the CCl_4_-induced oxidative stress significantly (*p* < 0.05) by scavenging the reactive oxygen species and enhancing the cellular antioxidant indices. In addition, these extracts significantly (*p* < 0.05) reduced the CCl_4_-induced inflammation by inhibiting the gene expression of NF-кB, iNOS, and in turn the level of nitric oxide. Consequently, the morphological appearance of Vero cells, cellular necrosis, and the gene expression of kidney injury molecule-1 (a marker of renal injury) after these treatments were improved. The AVE improved CCl_4_-induced oxidative and inflammatory stress in Vero cells and showed a more potent effect than the commonly used alpha-Ketoanalogue drug (ketosteril) in most of the studied assays.

**Conclusion:**

Thus, the studied plant extracts, especially AVE can be considered as promising extracts in the management of nephrotoxicity and other chronic diseases associated with oxidative stress and inflammation.

**Graphical abstract:**

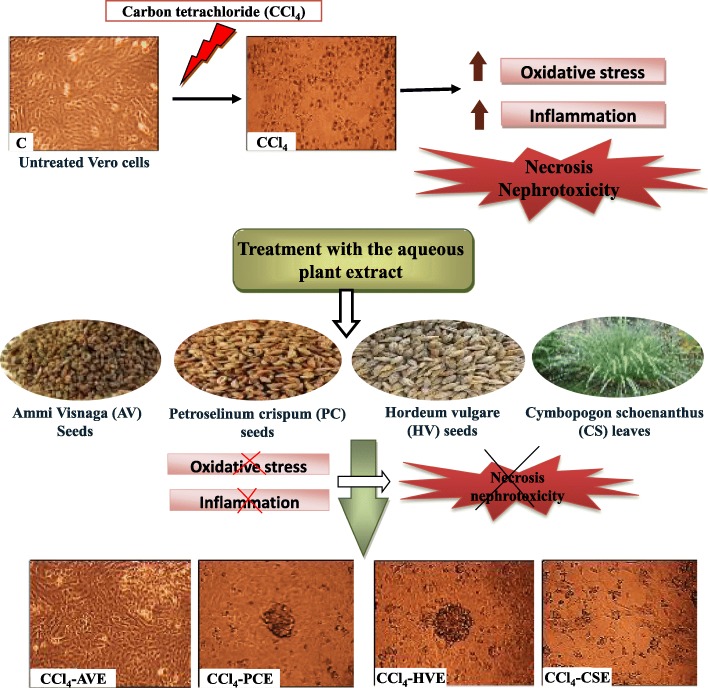

## Background

The kidney is an essential organ required by the body to perform several important functions including the maintenance of homeostasis, detoxification, and excretion of toxic metabolites and drugs. Constant exposure to drugs or chemical reagents jeopardizes the kidneys to nephrotoxicity, which is one of the most common renal problems. Aspirin (analgesic), Fluoxetine (antidepressant), Diphenhydramine (antihistamine), and Adefovir (antiretroviral) can cause nephrotoxicity [1]. Also, some halogenated hydrocarbons like carbon tetrachloride (CCl_4_) that is commonly used in the chlorofluorocarbons synthesis, anthelmintics, and grain fumigants can induce nephrotoxicity [2]. This kind of toxicity is associated with altered intraglomerular hemodynamics, tubular cell toxicity, inflammation, crystal nephropathy, rhabdomyolysis, and thrombotic microangiopathy. All of these damage effects will lead to sodium and water retention, hyperkalemia, metabolic acidosis, glomerulonephritis, and reduction in glomerular filtration rate [1]. The available treatments for patients with nephrotoxicity depend on the use of synthetic drugs that cause many side effects. Also, the prolonged protein restriction with the use of alpha-Ketoanalogue drugs such as Ketosteril (Ks) must be followed. These drugs could improve the nutritional deficiencies caused by protein-restricted diets due to the ability of their components to get converted in the body into essential amino acids [3]. In addition, alpha-Ketoanalogues delay the initiation of dialysis in chronic nephropathic patients due to their efficient ability to reduce the blood urea and ammonia levels. However, these medications act only as dietary supplements and are unable to preserve the kidney function [4]. Therefore, healthcare professionals continuously seek alternative therapies such as herbal remedies owing to their efficiency, availability, and fewer side effects [5].

Ethnomedicinal plants have many advantages over prescription medications or traditional medicine. They are capable of synthesizing thousands of diverse bioactive constituents including alkaloids, terpenoids, carotenoids, isoprenoids, flavonoid, phenolic acids, phytoestrogens, etc. These molecules may function as antioxidants, anticarcinogenic, hypoglycemic, and anti-inflammatory agents making medicinal plants good candidates for treating several diseases including kidney damage [6]. *Ammi visnaga* (AV), *Petroselinum crispum* (PC), *Hordeum vulgare* (HV), and *Cymbopogon schoenanthus* (CS) have been establishing significant attention in treating several kidney stones with other disorders [7–13]. The AV is a species of flowering plant in the carrot family recognized by many common names, including bisnaga, toothpick weed, and khella. Europe, Asia, and North Africa are its native habitats. Ancient Egyptians used it as an herbal medicine for renal colic and one of its components (khellin) was used as a smooth muscle relaxant and has pleiotropic effects on urolithiasis [14]. The PC (parsley) belonging to the family Umbelliferae and it is widely distributed in Western Asia, Mediterranean, and several European countries. Various pharmacological activities, such as antioxidant, anti-inflammatory, diuretic, nephroprotective, anti-urolithiasis, anticancer, enzyme-modulator, and anti-hypertensive actions, have been reported for this plant [9]. The HV belongs to the family Gramineae or Poaceae and it is locally known as ‘Barley’ or ‘jav’. It has several medicinal activities such as the treatment of urinary stones as well as diabetes and hyperlipidemia [15]. The CS is a desert species that grows in dry stony places and it is commonly known as Lemongrass, Camel grass Sakhbar, Izkhir or Athkhar. This plant is well-known in Egyptian folk medicine due to its antioxidant, anti-inflammatory, detoxification, antispasmodic, anti-rheumatism, anti-anorexia, and chemoprotective properties. Oral intake of its water extract is used as a potent diuretic remedy against kidney stones in North African arid Sahara [7].

Based on the well-known efficiency of the AV, PC, HV, and CS against different kidney stones, the current study for the first time examined the efficiency of their water extracts against the CCl_4_-induced nephrotoxicity. In addition, this study compared the efficiency of these extracts with the currently used drug for nephropathic patients (Ks) to evaluate their degree of potency. Here, we used Vero cells (African green monkey kidney cells) as an in vitro model for the study. This due to now it has become crucial and commonly used the cell cultures to support the research prior to the studies on animals and the clinical trials on human. The anti-nephrotoxic roles of these extracts were evaluated by investigating their suppressing effects on CCl_4_-induced oxidative stress and inflammation (necrotic mediators). To explain these probable activities, the phytochemicals and antioxidant abilities of the extracts were examined.

## Methods

### Chemicals

Folin-Ciocalteau reagent, 4-hydroxycinnamic acid (4-HCA), catechin, quercetin (QR), Ursolic acid (UA), butylated hydroxytoluene (BHT), 2,2-azino-bis (3-ethylbenzthiazoline-6-sulfonic acid (ABTS), α, α-diphenyl-β-picrylhydrazyl (DPPH), CCl_4_, 2′,7′-Dichlorofluorescin diacetate (DCFH-DA) probe, Ethidium bromide (EB), acridine orange (AO), thiobarbituric acid (TBA), reduced glutathione (GSH), propidium iodide (PI), 3-(4,5-dimethylthiazol-2yl-)-2,5-diphenyl tetrazolium bromide (MTT), and tetramethoxypropane (TMP) were purchased from Sigma-Aldrich (St. Louis, MO, USA). The Ks drug was manufactured by Fresenius Kabi Company (Hamburg, Germany) where one film-coated tablet contains α-keto analogue of isoleucine, leucine, phenylalanine, valine, methionine with other amino acids including L-lysine, L-threonine, L-tryptophan, L-histidine, and L-tyrosine. Dulbecco’s Modified Eagle Medium (DMEM), fetal bovine serum (FBS), and trypsin were obtained from Lonza, USA. Gene JET RNA purification kit, cDNA synthesis kit, and 2X SYBR green master mix kit were supplied from ThermoScientific, USA. Primers for nuclear factor-kappa (NF-к) B and inducible nitric oxide synthase (iNOS) were purchased from Bioneer, Korea. Other chemicals were obtained with a high grade.

### Plant material and extraction

The seeds of AV (NCBI:txid1053409), PC (NCBI:txid4043), and HV (NCBI:txid4513), and the leaves of CS (NCBI:txid79841) were obtained from the local market in Egypt in the dried form. In Egypt, these plants are available in the supermarkets and in the herbal stores. Experts are present in these stores for plant identification and authentication. Each plant type was ground individually to obtain the powdered form that was passed through a 20-mesh sieve with a particle size of 0.9 mm. Then 50 g of each plant powder was extracted twice by soaking in 500 mL of autoclaved distilled water for 72 h at 25 °C with continuous shaking. Finally, the extract was filtered and lyophilized (Telstar, Terrassa, Spain) to get the powdered form (AVE, PCE, HVE, and CSE, respectively).

### Phytochemical content and HPLC analysis for phenolics

Polyphenols including flavonoids (flavonols and anthocyanins) and tannins and triterpenoids were quantified in the four studied plant extracts. Total polyphenols (as HCA equivalents) were determined using the Folin-Ciocalteau method [16]. Flavonoid content was assessed spectrophotometrically using 10% AlCl_3_ and 5% sodium nitrite solutions and the concentration was calculated using catechin calibration curve [17]. Total flavonols were quantified using 50 g/l sodium acetate and 2% AlCl_3_ solutions [18], the absorbance was recorded at 440 nm and the concentration of total flavonols was calculated via a QR calibration curve. Anthocyanins were determined by the pH-differential assay depending on the sensitivity of these pigments to the change in the pH [19], the absorbance of the extract (A_e_) in two buffer solutions (pH 1.0 and pH 4.5) was read at 510 and 700 nm and calculated using the equation: [A_e_ = (A_510_ ˗ A_700_) _pH 1.0_ ˗ (A_510_ ˗ A_700_) _pH 4.5_], anthocyanins concentration as cyanidin-3-glucoside (Cy-3-glc) equivalent was quantified using the equation: [anthocyanin pigment = (A_e_ × MW × DF × 1000)/(ε × extract weight)]. The abbreviations, MW and ε refer to the molecular weight and the Cy-3-glc molar absorptivity, respectively whereas DF is the extract dilution factor. Total tannins were determined colorimetrically using catechin standard curve [20]. Triterpenoids content in each extract was quantified using vanillin color reaction and UA standard curve [21].

For HPLC analysis, 20 μl of each extract (AVE, PCE, HVE, or CSE) were separated on Zorbax Eclipse plusC18 column (100 mm × 4.6 mm, Agilent Technologies, Palo Alto, CA, USA). The separation was achieved at 284 nm using acetonitrile, 0.2% H_3_PO_4_, and methanol by ternary linear elution gradient. Under the same chromatographic conditions, pure phenolic standards were run to match the retention items [22].

### Vitamin C content

The concentration of vitamin C was quantified in each extract using 2,4 dinitrophenylhydrazine (2,4 DNPH) and standard vitamin. Each extract was deproteinized then incubated with a mixture of 2,4-DNPH (3%), CuSO4 (0.05%), thiourea (0.4%), and H_2_SO_4_ (65%) for 1.5 h at 37 °C. At the end of the incubation period, H_2_SO_4_ was added and the absorbance of the colored solution was read at 520 nm after 30 min.

### Antioxidant activities

The total antioxidant capacity (TAC) and antiradical potentials (anti- ABTS^+^, DPPH, and NO radicals) of the studied extracts (AVE, PCE, HVE, and CSE) were used to evaluate their antioxidant effects. Evaluation of the antiradical effect of each extract was done using the IC_50_ value (50% inhibitory concentration).

A mixture of 4 mM ammonium molybdate, 28 mM sodium phosphate, and 0.6 M H_2_SO_4_ was used to determine the TAC of the studied extracts. The colored product that was produced from the reduction of phosphomolybdate by each extract in 95 °C (90 min) was measured at 695 nm using BHT as a standard antioxidant [23].

The ability of the extracts to reduce the ABTS^+^ radical to ABTS can be indicated by the fading in the radical blue-green color using the ABTS^+^ radical cation-decolorization method [24]. ABTS^+^ radical was prepared by incubating 7 mM ABTS with 140 mM potassium persulphate in dark for 16 h at 25°C before mixing with each extract or standard antioxidant (BHT). The absorbance of the remaining blue color was measured at 734 nm for calculating the ABTS^+^ radical % inhibition. Also, the DPPH scavenging capability of the studied extracts was determined by reading the absorbance of the non-scavenged radical at 490 nm [25]. Griess reaction using Griess reagent (0.1% naphthylethylenediamine dihydrochloride, 1% sulfanilamide, and 2% phosphoric acid) and sodium nitroprusside evaluated the NO scavenging activity of the extracts [26], the reaction gave colored azo dye (bright-reddish-purple), its absorbance was measured at 490 nm.

### Renal cell culture and cytotoxicity assay

The African green monkey (*Cercopithecus aethiops*) renal epithelial cells (Vero, American Type Culture Collection “ATCC”, CCL-81, USA) were maintained in DMEM containing 5% FBS. After seeding 6000 cells per well of 96 well cell culture plates and allowing cells to attach for 24 h, serial concentrations (2, 1, 0.5, 0.25, 0.125 mg/mL) of each extract were added. Then plates were incubated for 72 h at 37 °C in 5% CO_2_ incubator. The cytotoxicity of each investigated extract was detected using MTT assay [27] by adding 20 μl of MTT (5 mg/mL) to each well followed by incubating the plates for 3 h. After removing MTT, 100 μl of DMSO was added and the absorbance was measured with a microplate reader (BMG LabTech, Germany) at 570 nm. The concentrations corresponding to 50 and 100% cell viability (IC_50_ and EC_100_, respectively) were determined by the GraphPad Instat software.

### Development of the nephrotoxicity in vitro model and determination of the anti-nephrotoxicity effective dose of each studied extract

After 24 h of renal cells seeding in 96 well cell culture plates, they were exposed to 0.13 mM CCl_4_ for 72 h to induce nephrotoxicity according to the method of Abu-Serie and Habashy [22]. The damaged renal cells were treated with different concentrations of the tested extracts and then incubated at 37 °C for 72 h in 5% CO2 incubator. The percentage of cell viability in the untreated and treated damaged cells in comparison with the healthy ones were determined by MTT assay as described above. The effective dose of each extract, which alleviated the damage in the renal cells by 100% (ED_100_) was calculated using GraphPad Instat software. The effective dose of each tested extract and for Ks was used for detection of renal necrotic cells, oxidative stress parameters, and gene expression. Moreover, morphological changes before and after treatment of the damaged renal cells were investigated using a phase contrast microscope (Olympus, Japan).

### Detection of renal necrotic cells

Cells were seeded in 6-well cell culture plate and treated with CCl_4_ for nephrotoxicity induction, and then incubated in 5% CO_2_ incubator (37 °C) with the ED_100_ value for each of AVE, PCE, HVE, CSE, or Ks for 72 h. Subsequently, cells were stained with double nuclear dyes for detection of necrotic renal cell populations using a fluorescence microscope and flow cytometry. Normal untreated cells (negative control) and CCl_4_-treated cells (positive control) with no further treatments were included in both assays.

Cells in the microtiter plate were stained with 100 μg/mL of EB and AO dyes and then observed under the fluorescent phase contrast microscope (Olympus, Japan). In addition, cells of other plates were trypsinized and incubated for 15 min with annexin V/PI; afterwards cells were fixed and incubated for 15 min with streptavidin-fluorescein (5 μg/mL). The flow cytometry (Partec, Germany) was used for PI-stained necrotic cell population quantification using the phycoerythrin emission signal detector (FL2) against annexin-FITC emission signal detector (FL1).

### Assessment of the oxidative stress parameters

#### Determination of intracellular ROS level

The extremely sensitive DCFH-DA fluorescent probe was used for the assessment of the ROS level [28]. Renal cells were preloaded for 30 min with DCFH-DA (5 μM) at 37 °C. Then the DCF fluorescent molecules were liberated after ROS oxidation of the cellular esterases cleaved the non-fluorescent product (H_2_DCF) from DCFH-DA. The fluorescence intensity was determined by the flow cytometry at 488 nm (excitation) and 530 nm (emission) wavelengths.

#### Lipid peroxidation assay

The amount of the lipid peroxidation was determined using TBA reactive substances (TBARS) colorimetric method [29]. The method is based on the reaction of malondialdehyde (the decomposition product of lipid peroxides) with TBA (0.67%) in boiling water bath. The produced chromophore was read at 532 nm and the concentration of the lipid peroxidation was calculated using the calibration curve of TMP standard.

#### Antioxidant indices and total protein levels

The antioxidant indices including the GSH levels as well as the superoxide dismutase (SOD) and glutathione peroxidase (GPX) activities were determined. Ellman’s reagent (5, 5′-dithio bis2- nitrobenzoic acid) was used for determination of the GSH content. The absorbance of the produced yellow-colored product was read at 412 nm [30]*.* The activity of Cu/Zn SOD was determined by pyrogallol autooxidation method [31]. The absorbance change for 2 min was recorded at 420 nm. The unit of activity is described by the amount of enzyme that suppresses 50% of the pyrogallol (20 mM) autooxidation rate under the standard conditions. The GPX activity was assessed colorimetrically following the method of Rotruck [32] using cumene hydroperoxide and GSH as enzyme substrates. Protein content was assessed using Bradford Coomassie brilliant blue assay [33]. The obtained blue colored complex was read at 630 nm and the protein concentration was calculated from the standard curve of the bovine serum albumin.

#### Determination of the inflammatory mediators and kidney injury molecule-1 (KIM-1)

After 72 h of treating cells with AVE, PCE, HVE, and CSE, cells were centrifuged and the supernatant was collected and used for colorimetric determination of NO by nitrite using Griess reaction [26]. While, the cells were used for RNA extraction and quantification of NF-кB, iNOS, and Kim-1.

Total RNA was extracted from untreated and CCl_4_-treated, extract-treated and Ks-treated cells using Gene JET RNA Purification Kit by following the manufacturer’s protocol. The concentration and purity of the obtained RNAs were measured using an UV-spectrophotometer. Each RNA sample was used in the preparation of cDNA by reverse transcriptase-polymerase chain reaction (RT-PCR, Qiagen, Germany) via the cDNA Synthesis Kit. The gene expression levels of the β-actin (reference gene) and target genes were measured by RT-PCR using the gene-specific primers (forward and reverse). The following primers were used: NF-кB, forward 5′-ATGGCTTCTATGAGGCTGAG-3, reverse: 5′-GTTGTTGTTGGTCTGGATGC-3′; iNOS, forward: 5′-GTTCTCAAGGCACAGGTCTC-3′, reverse: 5′-GCAGGTCACTTATGTCACTTATC-3′; KIM-1, forward:5′-TGGCACTGTGACATCCTCAGA-3′; reverse: 5′-GCAACGGACATGCCAACATA-3′ and β-actin, forward: 5′-AAGCAGGAGTATGACGAGTCCG-3′, reverse: 5′-GCCTTCATACATCTCAAGTTGG-3′.

The reaction mixture contained 50 ng cDNA template, 12.5 μL of a 2X SYBR green master mix, 0.3 μL of 10 μM forward primer, 0.3 μL of 10 μM reverse primer and the volume was completed to 25 μL with nuclease-free water. The qPCR program was applied as following, enzyme activation (one cycle at 95 °C for 15 min) followed by 40 cycles of denaturation (95 °C for 15 s), annealing (60 °C for 1 min) and extension (72 °C for 30 s). The target genes expression was calculated using the comparative Ct method (the number of threshold cycle at cross-point between threshold and amplification plot). The target genes CT values were normalized to that of β-actin according to manufacturer’s instructions.

### Statistical analysis

The data are expressed as mean ± SE and the significant values were considered at *p* < 0.05. One-way analysis of variance (ANOVA) by Duncan’s test was used for evaluating the difference between the mean values of the studied treatments. The analysis was done for three measurements using SPSS software version 16. The EC_100_, IC_50_, and ED_100_ values were calculated by GraphPad Instate software version 3. Heat map plots were generated by ClustVis web tool (https://biit.cs.ut.ee/clustvis/) [34].

## Results

### Characterization of the studied extracts

**Fig. 1:**
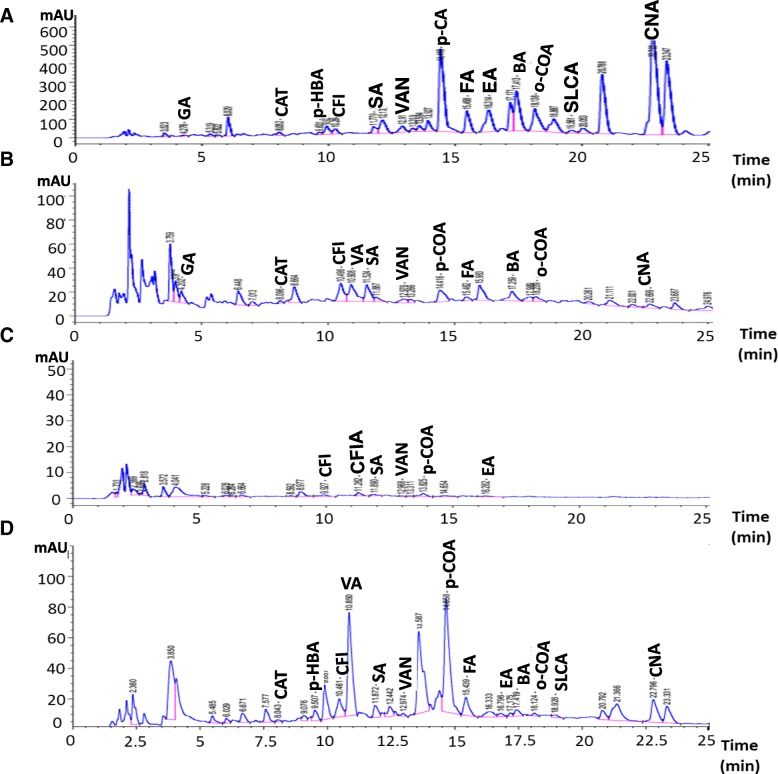
HPLC chromatograms of the phenolic compounds in (**a**) *Ammi visnaga* extract (AVE*)* (**b**) *Petroselinum crispum* extract (PCE) (**c**) *Hordeum vulgare* extract (HVE) and (**d**) *Cymbopogon schoenanthus* extract (CSE). *CAN; Cinnamic acid, CAT; catechol, CFI*; *caffeine*, *CFIA; Caffeic acid, COA*; coumaric acid, *EA; Ellagic acid, FA; Ferulic acid, GA; gallic acid, p-HBA*; p-hydroxybenzoic acid, *SA, Syringic acid, SLCA; Salicylic acid, VA*; vanillic acid, *VAN; Vanillin*

The extraction yield of each of the studied extracts is presented in Table 1 where the lowest value was detected for AVE. Table 1 also elucidated the chemical composition of the extracts and the results showed that each one contains considerable quantities of phenolics, flavonoids, anthocyanins, flavonols, tannins, triterpenoids, and vitamin C. As seen in the Table, AVE contains a significant (*p* < 0.05) high amount of phenolic compounds (flavonoids, flavonols, and tannins), while, the triterpenoids content of PCE was significantly (*p* < 0.05) high. Whereas CSE contains a significant (*p* < 0.05) high vitamin C. The HPLC analysis identified different phenolic compounds in the studied extracts by comparing their specific retention time with that of known phenolic standards (Table 1). Hence, in which thirteen phenolic compounds were detected in each of AVE and CSE (Fig. 1a and d, respectively), eleven ones in PCE (Fig. 1b), and six ones in HVE (Fig. 1c).

**Table 1:** The yield and chemical composition of the studied plant extracts

Extracts yield and composition	*Ammi visnaga* extract (AVE)	*Petroselinum crispum* extract (PCE)	*Hordeum vulgare* extract (HVE)	*Cymbopogon schoenanthus* extract (CSE)
Yield (g%)	8.28 ± 0.88^c^	17.86 ± 0.74^a^	12.27 ± 0.44^b^	10.93 ± 0.50^b^
Phenolics (mg 4-HCA eq/g extract)	366.42 ± 3.89^a^	99.84 ± 28.11^c^	248.16 ± 5.82^b^	210.35 ± 31.99^b^
Flavonoids (mg catechin eq/g extract)	260.42 ± 3.46^a^	30.86 ± 0.89^b^	9.64 ± 0.10^c^	9.645 ± 0.34^c^
Anthocyanins (mg Cy-3-glc eq/g extract)	0.75 ± 0.08^c^	0.59 ± 0.09^d^	1.08 ± 0.08^a^	1.04 ± 0.12^b^
Flavonols (mg QR eq/g extract)	3.92 ± 0.00^a^	1.49 ± 0.71^b^	0.98 ± 0.19^b^	0.20 ± 0.08^b^
Tannins (mg catechin eq/ g extract)	192.50 ± 27.50^a^	76.25 ± 0.00^b^	41.87 ± 1.87^b^	43.75 ± 10.00^b^
Triterpenoids (mg UA eq/ g extract)	0.76 ± 0.08^b^	1.78 ± 0.04^a^	0.45 ± 0.02^c^	0.34 ± 0.02^c^
Vitamin C (mg/g extract)	0.75 ± 0.01^d^	4.16 ± 0.50^b^	2.18 ± 0.02^c^	11.44 ± 0.10^a^
Phenolics, mg/g extract (RT, min)				
Gallic acid (4.276)	0.04 ± 0.00 ^b^	0.06 ± 0.00 ^a^	ND	ND
p-Hydroxybenzoic acid (9.631)	0.43 ± 0.01 ^a^	0.04 ± 0.00 ^b^	ND	0.03 ± 0.00 ^c^
Caffeine (10.264)	0.28 ± 0.00 ^a^	ND	ND	0.18 ± 0.00 ^b^
Vanillic acid (10.923)	0.28 ± 0.00 ^a^	0.18 ± 0.00 ^b^	0.01 ± 0.00 ^d^	0.13 ± 0.00 ^c^
Caffeic acid (11.230)	ND	0.23 ± 0.01 ^b^	ND	0.67 ± 0.00 ^a^
Syringic acid (11.779)	ND	ND	0.01 ± 0.00	ND
Vanillin (12.911)	0.27 ± 0.00 ^a^	0.12 ± 0.00 ^b^	0.01 ± 0.00 ^d^	0.06 ± 0.00 ^c^
p-Coumaric acid (14.418)	0.26 ± 0.02 ^a^	0.03 ± 0.00 ^b^	0.001 ± 0.00 ^d^	0.01 ± 0.00 ^c^
Ferulic acid (15.458)	1.73 ± 0.01 ^a^	0.05 ± 0.00 ^c^	0.01 ± 0.00 ^c^	0.30 ± 0.04 ^b^
Catechol (8.052)	0.77 ± 0.03 ^a^	0.02 ± 0.00 ^c^	ND	0.09 ± 0.00 ^b^
Ellagic acid (16.314)	3.09 ± 0.00 ^a^	ND	0.01 ± 0.00 ^b^	0.04 ± 0.00 ^b^
Benzoic acid (17.413)	24.89 ± 1.64 ^a^	1.17 ± 0.04 ^b^	ND	0.47 ± 0.00 ^c^
o-Coumaric acid (18.138)	0.70 ± 0.01 ^a^	0.03 ± 0.00 ^b^	ND	0.01 ± 0.00 ^b^
Salicylic acid (19.561)	0.43 ± 0.04 ^a^	ND	ND	0.04 ± 0.00 ^b^
Cinnamic acid (22.793)	3.65 ± 0.16 ^a^	0.02 ± 0.00 ^c^	ND	0.08 ± 0.00 ^b^

Results are presented as Mean ± SE (*n* = 3). Different letters in the same row are significantly different at *p* < 0.05. *Cy-3-glc*, cyanidin-3-glucoside, *4-HCA* 4-hydroxycinnamic acid*, QR* quercetin, *RT* retention time, *ND* not detected

### Antioxidant activities of the studied extracts

**Fig. 2:**
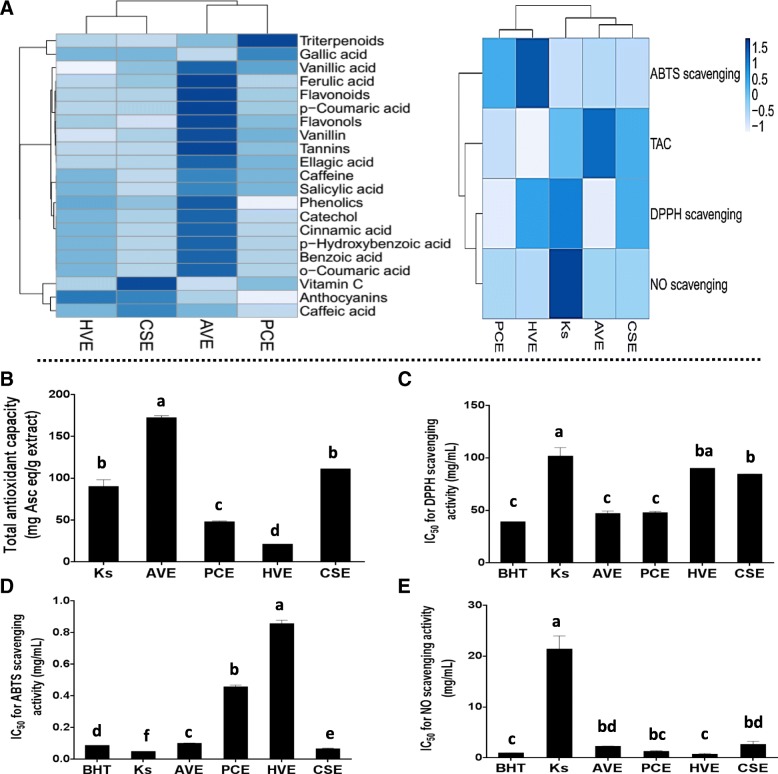
Antioxidant activities of *Ammi visnaga* (AV), *Petroselinum crispum* (PC), *Hordeum vulgare* (HV), and *Cymbopogon schoenanthus* (CS) extracts in comparison with the Ketosteril (Ks) and butylated hydroxytoluene (BHT). (**a**) Heat map distribution of the phytochemical content and in vitro antioxidant activities of the four studied extracts, the color distributed from white (low concentrations) to blue (high concentrations) (**b**) Total antioxidant capacity (**c**) α, α-Diphenyl-β-picrylhydrazyl (DPPH) scavenging activity (**d**) 2,2-azino-bis (3-ethylbenzthiazoline-6-sulfonic acid (ABTS) scavenging activity (**e**) Nitric oxide (NO) scavenging activity. Results are presented as mean ± SE (*n* = 3). Different letters for the same parameter are significantly different at *p* < 0.05

The results of the antioxidant activities of the studied extracts are presented in Table 1 and Fig. 2, which reveals a variety in the antioxidant activities among the extracts. The TAC data (Fig. 2b) show that AVE has the strongest capacity followed by HVE then CPE and PCE. The Ks exhibited an antioxidant capacity similar in its potency to HVE, but less than that of AVE. The antiradical screening of the tested extracts showed that the ability to scavenge DPPH by AVE and PCE was the highest with potency equal to that of BHT. Whereas HVE and CSE exhibited the lowest scavenging activity, yet the DPPH scavenging activity of all of the studied extracts remained significantly (*p* < 0.05) more potent than that of Ks (Fig. 2c). Regarding ABTS scavenging ability, all the studied extracts were significantly (*p* < 0.05) less potent than Ks with CSE having the highest scavenging ability allowing it to become as potent as BHT (Fig. 2d). Concerning the NO scavenging ability, Ks was lower than that of all the studied extracts and BHT, on the other hand, only PCE and HVE had the same efficiency as the BHT (Fig. 2e).

### Vero cell viability and cytotoxic effect of the studied extracts

The results in Table 2 reveal that the studied extracts varied in their safety upon Vero cells, but are safer than Ks, the order of their safety from highest to the lowest is HVE > PCE > CSE > AVE. The Table also presents the IC_50_ and ED_100_ values of the studied extracts, which demonstrates the highest potency of AVE and the lowest efficiency of the Ks.

**Table 2:** Effect of the studied plant extracts and Ketosteril (Ks) on the viability of the untreated Vero cells and their effective doses (ED_100_) that produced 100% therapeutic response from the carbon tetrachloride (CCl_4_)-induced nephrotoxicity

	*Ammi visnaga* extract (AVE)	*Petroselinum crispum* extract (PCE)	*Hordeum vulgare* extract (HVE)	*Cymbopogon schoenanthus* extract (CSE)	Ks
EC_100_ (μg/ml)	999.48 ± 49.05 ^d^	2209.87 ± 23.96 ^b^	2709.07 ± 18.20 ^a^	1371.75 ± 7.65 ^c^	450.74 ± 1.31 ^e^
IC_50_ (μg/ml)	6334.53 ± 86.20 ^a^	9023.38 ± 46.36 ^b^	9024.16 ± 611.32 ^b^	11,725.59 ± 13.40 ^c^	12,785.56 ± 1.24 ^d^
ED_100_ (μg/ml)	83.64 ± 3.06 ^a^	140.71 ± 1.78 ^b^	174.77 ± 2.06 ^cd^	148.10 ± 14.42 ^bc^	189.05 ± 5.74 ^d^

Results are presented as Mean ± SE (*n* = 3). Different letters in the same row are significantly different at *p* < 0.05. *EC*_*100*_, safe concentrations of the studied extracts that caused 100% viability for Vero cells; *IC*_*50*_, concentration of the studied extracts that caused 50% viability for Vero cells

### Induction of nephrotoxicity by CCl_4_ and the ameliorating effects of the studied extracts

The results of the current study revealed that CCl_4_ induced necrosis and injury in Vero cells through disturbing cellular redox state (oxidative stress) and inducing inflammation. The treatments with the four-studied plant extracts (AVE, PCE, HVE, and CSE) improved these disturbances by different potencies, where their effect was compared with Ks.

### Improving CCl_4_-induced oxidative stress by the studied extracts

**Fig. 3:**
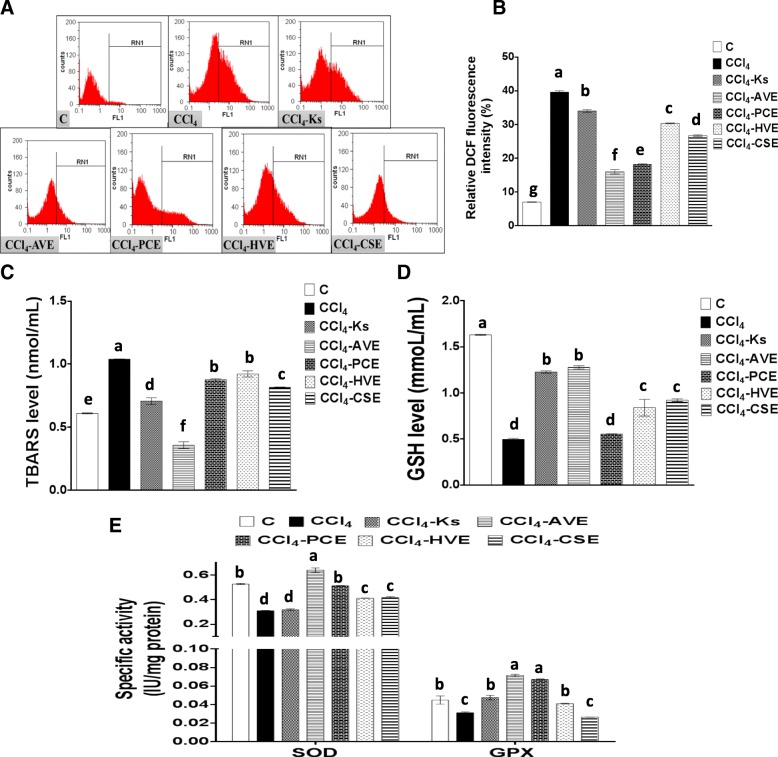
Effect of the studied extracts on the carbon tetrachloride (CCl_4_)-induced oxidative stress in Vero Cells in comparison with the Ketosteril (Ks). (**a**) Flow cytometric analysis of ROS production in Vero cells using dichlorofluorescein (DCF) (**b**) Quantification of the DCF flow cytometric data (**c**) Thiobarbituric acid reactive substances (TBARS) level (**d**) Reduced glutathione (GSH) level (**e**) Superoxide dismutase (SOD) and glutathione peroxidase (GPX) activities. *C*; the untreated control cells, *AVE*, *Ammi visnaga* extract; *PCE*, *Petroselinum crispum* extract; *HVE*, *Hordeum vulgare* extract; *CSE*, *Cymbopogon schoenanthus* extract. Results are presented as mean ± SE (*n* = 3). Different letters for the same parameter are significantly different at *p* < 0.05

The exposure of Vero cells to CCl_4_ resulted in an elevation of both ROS (467.62%) and lipid peroxidation (TBARS, 70.18%) levels. This was accompanied by a depletion in GSH level (6.55%) along with the activities of SOD (41.08%) and GPX (30.51%). These results indicate the induction of the oxidative stress state in Vero cells by the CCl_4_ toxicant. Each of the four studied extract treatments was able to ameliorate this damaging effect and efficiently preserve the balance between ROS and the redox system in Vero cells (Fig. 3). From Fig. 3a, b, the order of decreasing the DCF emission can be outlined as following AVE (59.67%) > PCE (54.16%) > CSE (32.66%) > HVE (23.50%). The efficiency of these extracts was significantly (*p* < 0.05) higher than Ks (14.23%), which had the highest DCF emission value. The decrease in ROS level has been associated with a decrease in the level of TBARS (Fig. 3c), which was dramatically decreased after the treatment with AVE (65.53%) more than the other extracts (11.28–21.48%). Referring to Ks potency in decreasing the TBARS level (31.89%), it was found to be less potent than AVE, but higher than the other extracts. Depletion in the ROS and TBARS levels by the studied extracts had a great impact on the antioxidant indices levels. Hence, the level of the GSH (Fig. 3d) and the activities of the antioxidant enzymes (Fig. 3e) were recovered after the extract treatments with the highest effects belonging to AVE. The GSH level elevation corresponding to the different treatments are as follows 157.43% (AVE), 85.80% (CSE), 69.53% (HVE), and 11.72% (PCE). Comparing with Ks potency in boosting GSH level (147.21%), AVE had the same potency, but the other tested extracts had less potency.

Regarding the antioxidant enzymes activities, AVE was found to be the most potent studied extract in restoring SOD and GPX activities (105.99 and 129.17%, respectively) with a potency extremely higher than the Ks (2.45 and 52.24%, respectively). Also, PCE treatment was able to up-regulate the activities of these enzymes by 65.11 and 115.06%, respectively, while HVE elevated them only by 32.63 and 31.41%, respectively and CSE by 34.54 and 15.06%, respectively.

### Reduction of the CCl_4_-induced inflammatory mediators by the studied extracts

**Fig. 4:**
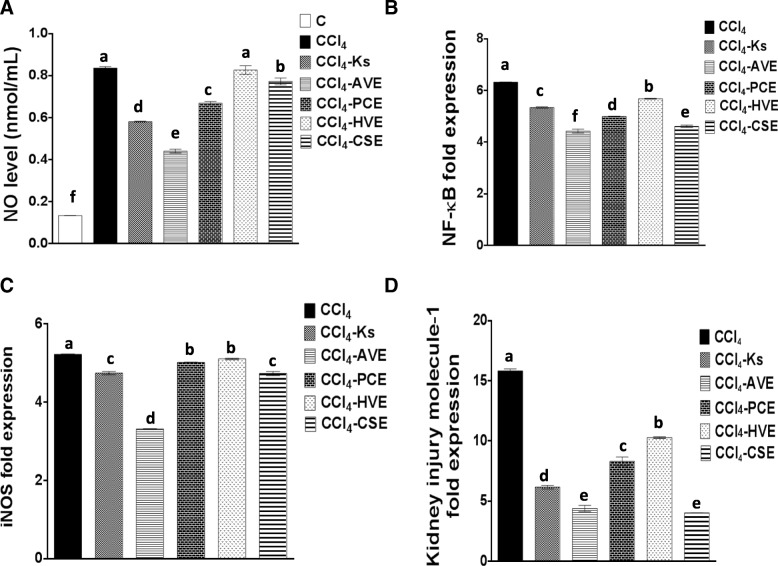
Effect of the studied extracts on the carbon tetrachloride (CCl_4_)-induced inflammation and injury in Vero Cells in comparison with the Ketosteril (Ks). (**a**) Nitric oxide (NO) level (**b**) Nuclear factor (NF) кB gene expression (**c**) Inducible nitric oxide synthase (iNOS) gene expression (**d**) Kidney injury molecule-1 gene expression. *C*; the untreated control cells, *AVE*, *Ammi visnaga* extract; *PCE*, *Petroselinum crispum* extract; *HVE*, *Hordeum vulgare* extract; *CSE*, *Cymbopogon schoenanthus* extract. Results are presented as mean ± SE (*n* = 3). Different letters for the same parameter are significantly different at *p* < 0.05

Graph A-C in Fig. 4 shows that incubation of Vero cells with CCl_4_ for 72 h induced inflammation through raising the gene expression of inflammatory mediators (NF-кB and iNOS) and the level of NO. Treatments with each of the four studied extracts exhibited an anti-inflammatory role by a significant (*p* < 0.05) depletion in these inflammatory mediators. The results showed that CCl_4_ generated an elevation in NO level by 527.50% which was significantly (*p* < 0.05) reduced after treating with AVE, PCE, and CSE by 47.27, 19.97, and 7.54%, respectively, however, the HVE treatment resulted in a non-significant (1.04%) reduction in NO level as compared with the CCl_4_-treated cells. Comparing with Ks (30.46%) in reducing NO level, AVE potency was higher, whereas the other three extracts showed lower efficiency. In addition, the gene expression of NF-кB and iNOS were significantly (*p* < 0.05) depleted in response to the different studied extract treatments, the highest percentage of depletion was observed with AVE (29.93 and 36.47%, respectively) followed by CSE (27.00 and 9.41%), PCE (21.01 and 3.93%), then HVE (10.09 and 2.25%). On the other hand, Ks had a less anti-inflammatory effect where it significantly (*p* < 0.05) reduced the gene expression of both NF-кB and iNOS by only 15.49 and 9.18%, respectively. These results elucidate the high anti-inflammatory potential of AVE over Ks and the other studied extracts.

### Induction of Vero cells necrosis by CCl_4_ and the anti-necrotic effect of the studied extracts

**Fig. 5:**
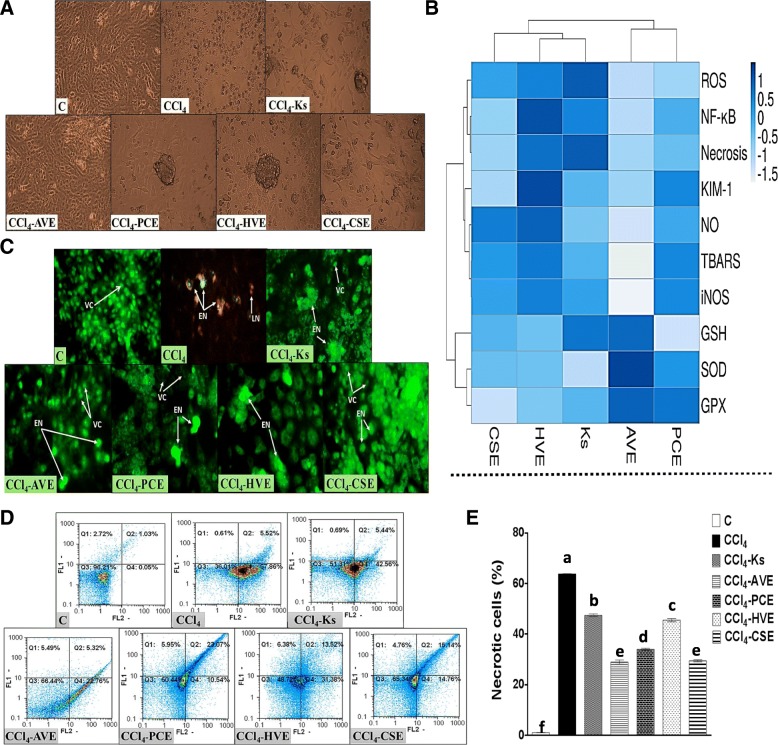
Microscopic investigation and flow cytometric analysis for the carbon tetrachloride (CCl_4_)-induced necrosis in Vero Cells and the anti-necrotic effect of the studied extracts in comparison with the Ketosteril (Ks). (**a**) Photomicrographs taken by the inverted microscope (**b**) Heat map distribution of the necrotic mediators, the color distributed from white (low level/expression or activity) to blue (high level/expression or activity) (**c**) Nuclear double staining of Vero cells using acridine orange (AO)/ethidium bromide (EB) (**d**) Annexin V/Propidium Iodide (PI) flow cytometry charts (**e**) Quantification of the necrotic cell populations, results are presented as mean ± SE (*n* = 3). Different letters for the same parameter are significantly different at *p* < 0.05. *C*; the untreated control cells, *AVE*, *Ammi visnaga* extract; *PCE*, *Petroselinum crispum* extract; *HVE*, *Hordeum vulgare* extract; *CSE*, *Cymbopogon schoenanthus* extract; *VC*, viable cells; *EN*, Early necrotic cells; *LN*, Late necrotic cells

As observed under the inverted microscope (Fig. 5a), the untreated Vero cells grew in a monolayer with high confluency and showed the typical elongated fibroblast-like cells. After the treatment with CCl_4_, cells experienced a decrease in confluency with a spheroidal shape and swollen appearance indicating cell necrosis and loss of surface adhesion. After treatment with the plant extracts under investigation, the typical fibroblast-like morphology of the Vero cells was reestablished, and the number of damaged cells differed according to the potencies of these treatments. Treatment with AVE was able to restore most of the normal features of Vero cells more than the other plant extracts and Ks. These results were confirmed by the morphological appearance of Vero cells under the fluorescence microscope after their dual staining with AO/EB (Fig. 5c). Nuclei of the viable Vero cells can only be stained with AO and so they appeared with green fluorescence. Exposure of Vero cells to CCl_4_ induced cell membrane damage facilitating the diffusion of EB into the cells and staining their nuclei in the early necrotic stage with bright greenish-yellow color and in the late stage with red color. Treatment with the studied plant extracts improved the morphological appearance of the Vero cells which can be observed by the decline in the early necrotic cell population with the absence of the late necrotic cells. In addition, some packs of viable cells were visualized. AVE treatment was more efficient in restoring Vero cells morphological appearances than the other three plant extracts and Ks. In harmony with the morphological results, the annexin V/PI flow cytometric analysis (Fig. 5d, e) revealed an elevation in the percentage of Vero necrotic cell populations after exposure to CCl_4_ (5860.75%). However, the treatment with the studied extracts dramatically reduced the number of these cell populations, where the studied extracts exhibited a potency significantly (*p* < 0.05) higher than the that of Ks (25.56%). The most effective extracts were AVE (54.61%) and CSE (53.81%) followed by PCE (46.64%) then HVE (28.65%).

### Depletion of KIM-1 gene expression by the studied extracts

The anti-necrotic potential of the extracts under investigation was confirmed by the results of the KIM-1 gene expression (Fig. 4d). The exposure to CCl_4_ was significantly (*p* < 0.05) up-regulated the gene expression of this protein. Whereas treatment with the studied extracts as well as the standard drug dramatically reduced this expression. In harmony to the morphological and the flow cytometric results, AVE was shown to be the most effective in reducing the KIM-1 gene expression by 72.29% and in turn diminishing necrosis. The other three extracts (PCE, HVE, and CSE) significantly (*p* < 0.05) reduced its expression by 47.48, 35.05, and 74.5%, respectively. Comparing with Ks (61.01%), AVE and CSE had more reducing abilities while PCE and HVE had lower capabilities.

### Heat map analysis

The heat map chart summarizes the results of phytochemicals (Fig. 2a), in vitro antioxidant activities (Fig. 2a), and necrotic mediators (Fig. 5b). This diagram used the ClustVis tool for clustering the multivariate data values. The color of the chart [white (low) to blue (high)] was related to the concentration of the compound, IC_50_, or the level/expression of the parameter.

## Discussion

Nowadays, the use of traditional plants for improving healthcare has gained great interest. This may be related to the vital constituents of these plants, including phytochemicals, carotenoids, terpenoids, vitamins, and others. In the current study, the four tested plant extracts showed the presence of different quantities of phenolics, flavonoids, anthocyanins, flavonols, tannins, triterpenoids, and vitamin C. These constituents are known for their significance as antioxidants, anti-inflammatory, anti-aging, and anticancer agents. Therefore, the studied extracts may have an essential role in improving human health allowing these extracts to gain large medicinal importance in the future. The studied extracts were examined for their antioxidant activities and they exhibited different efficiencies as shown by the heat map diagram (Fig. 2a). In line with our results, the previous studies confirmed the antioxidant and antiradical potentials of different extracts from AV [35], PC, HV [9], and CS [15]. These antioxidant abilities may be owed to some of the extracts constituents such as phenolics (like phenolic acids, tannins, flavonoids “ flavonols and anthocyanins”), triterpenoids and vitamin C. These molecules are well known for their antioxidant activities and ROS/reactive nitrogen species (RNS) scavenging abilities. A large number of hydroxyl groups of the phenolic compounds are greatly essential, in which it influences their ROS/RNS scavenging abilities. In addition, these compounds are able to suppress the activity of ROS generating enzymes as well as upregulating antioxidant defense molecules [36]. The antioxidant potential of the phenolic compounds present in the studied extracts can be synergistically amplified in the presence of vitamin C [37]. This will aid in enhancing the scavenging ability and clearing ROS/RNS then decreasing their levels. In addition, the antiradical activities of several types of triterpenoids were reported previously [38]. Undoubtedly these antioxidant and antiradical abilities of the studied extracts provide them with an imperative use in human health and medicine fields.

The current study evaluated the cytotoxic effect of AV, PC, HV, and CS on the Vero cells, where the results revealed the high safety and efficiency of each extract especially AVE that was higher than the standard drug. This will increase the quality and importance of these extracts (particularly AVE) as an effective treatment for nephrotoxicity. In addition, the anti-nephrotoxic efficiencies of these extracts on the CCl_4_-exposed Vero cells were examined. The use of CCl_4_ as a nephrotoxicant was based on it being one of the well-known hepatotoxicants with a well-documented hepatotoxicity mechanism. Also, it can induce toxicity in many other tissues such as heart, kidney, brain, lung, testis, and blood [39]. Particularly, the kidney is a good target for CCl_4_ toxicity owing to its ability to harbor high amounts of CCl_4_ after systemic administration. The CCl_4_ can induce tissue toxicity through the excessive formation of free radicals and subsequently mediating oxidative stress. The kidney is highly vulnerable to oxidative stress damage as it is rich in mitochondria, which allows it to produce ROS from NADPH oxidases and mitochondrial respiratory chain [40]. The CCl_4_ is exposed to reductive dehalogenation during its metabolism by renal cytochrome P450 (CYP2E1) forming the reactive trichloromethyl-free radical (CCl_3_^•^). This reactive product will interact with molecular oxygen producing the trichloromethyl peroxy radical (CCl_3_OO^•^). The generated free radicals will either start peroxidative damage through binding to DNA and proteins or attack polyenoic fatty acids in cellular membranes giving secondary lipid radicals. Hence, initiating lipid peroxidation [41] will result in excessive production of ROS as well as consumption and reduction of GSH. The SOD is the first line of the cellular antioxidant defense system, which is essential for dismutation of the superoxide anion to H_2_O_2_ [31]. This is followed by GPX detoxifying H_2_O_2_ to water using GSH as a cofactor and a co-substrate [32]. Exposure of Vero cells to CCl_4_ caused a reduction in GSH level in concomitant with depletion in SOD and GPX activities. The loss of antioxidant enzymes activities may be related to the reduction in GSH level, which can inactivate GPX and result in H_2_O_2_ accumulation. High level of H_2_O_2_ can inhibit SOD activity raising superoxide anion radical that also inhibits the GPX activity [31]. Therefore, the reduction in GSH level with the loss of the antioxidant enzymes activities in Vero cells will accumulate more ROS exacerbating oxidative stress. These results are in line with the previous studies that confirm the induction of oxidative stress in this cell type after exposure to free radicals [42].

The present study showed varied potencies of AVE, PCE, HVE, and CSE on the damaging effect induced by CCl_4_. Hence, these extracts have the ability to reinstall the balance between ROS production and the cellular antioxidants (Fig. 5b). These observations are in correspondence with a few previous studies that were conducted on the alleviating power of these plants on oxidative stress and toxicity-induced by different toxicants other than CCl_4_ [43–46]. The present in vitro study confirms the potent antiradical activities of these extracts (Fig. 2), which may be related to their ingredients. Therefore, upon treating the CCl_4_-exposed Vero cells with each of these extracts, they scavenged the generated ROS and reduced its level (Fig. 3a, b). This will result in improving the cellular redox state and preventing ROS from damaging cellular biomolecules, which in turn reduced lipid peroxidation level. In addition, GSH and the antioxidant enzymes were preserved and their levels and activities were enhanced. This may be linked to the phytochemical constituents of the studied extracts such as phenolic acids, flavonoids, tannins, caffeine, catechol, and triterpenoids (Table 2). The importance of these compounds to plants resides in ameliorating oxidative stress which has been previously reported [47–51]. Moreover, vitamin C can attenuate renal injury in animal models by suppressing oxidative damage [52]. The present study showed that Ks was able to minimize the CCl_4_-induced oxidative stress in Vero cells, however AVE exhibited a higher efficiency to that of Ks as well as the three other studied extracts. This ultimate potency of AVE may be related to its higher levels of effective and functional constituents (phenolic acids, flavonoids, tannins, and flavonols) compared to the three other studied extracts (Table 1).

The ability of CCl_4_ to induce oxidative stress was the main cause for the elevation in the inflammatory mediators, and hence inflammation is the most common outcome of oxidative stress.. Our outcomes align with previous studies reporting the ability of CCl_4_ to activate the gene expression of various inflammatory markers, including NF-кB along with its activation [53]. The elevated levels of ROS generated by CCl_4_ is likely implicated in the activation of this pathway [54]. The NF-кB acts as a transcription factor controlling the expression of more than 500 genes related to inflammation as well as tumorigenesis and cellular survival/proliferation [55]. The NF-кB activates the expression of other inflammatory mediators such as iNOS, and TNF-α with others to amplify the inflammatory response [56]. Elevation of these inflammatory mediators amplifies oxidative stress within the cells; consequently the elevation of iNOS will lead to the production of more NO radicals. These radicals may interact with superoxide radicals yielding peroxynitrites with other RNS and increase the cellular damage [54]. Therefore, the production of inflammatory mediators is concomitant with oxidative stress and vice versa.

The ameliorating effects of the studied extracts over the CCl_4_-induced inflammation in Vero cells is possibly owed to the reduction in the ROS level along with the inhibition of the NF-кB pathway (Fig. 5b). In accordance with these findings, the anti-inflammatory roles of AV [57], PC [9], HV [58], and CS [59] was previously confirmed by few in vitro and in vivo studies. The presence of the phytochemicals (Table 1) in the studied extracts is of great essence by providing them with their powerful anti-inflammatory activities. As detected by the HPLC, these studied extracts contained certain types of phenolics, which were previously reported to inhibit the NF-кB expression and its related inflammatory mediators [60]. Polyphenols are also able to interact with and inactivate NF-кB and iNOS, subsequently modulating the production of NO and other inflammatory mediators [36]. In addition, the anti-inflammatory activities of flavonoids, tannins [61], triterpenoids [38], and vitamin C [52] through targeting the NF-кB pathway were reported before. The results showed the higher anti-inflammatory potential of AVE over the other studied extracts and Ks, which may be related to its higher phytochemical content.

The exposure of Vero cells to CCl_4_ may lead to renal necrosis as a result of the excessive ROS/RNS production that overwhelms the cellular scavenging ability. This condition may cause a passive mode of cell death (cell necrosis), which became implicated in increasing the inflammatory damage and exacerbating renal injury [36] as observed in Fig. 5. These outcomes support the existence of an interplay between oxidative stress, inflammation, and necrosis in kidney injury diseases [40]. As discussed above treatment with the studied extracts extremely diminished the oxidative and inflammatory stress. Therefore, the necrosis decreased, and the cells regenerated again (nearly restored their normal shape especially with AVE and CSE more than PCE and HVE). Hence, the antioxidants that scavenge ROS or boost the cellular antioxidant pool can have a therapeutic role in acute kidney injury [52]. KIM-1 is a type-1 transmembrane protein that acts as an adhesion molecule tethering cells to the extracellular matrix and interconnecting cells to each other. This protein is undetectable in the normal kidney; however, it is extremely expressed as a result of exposure to nephrotoxins. Thus, KIM-1 is considered as a sensitive biomarker for renal injury. The generated protein is extensively localized on the apical membrane of the proximal tubule where the tubule is mostly affected. KIM-1 confers epithelial cells the ability to recognize damaged cells and transform them into semi-professional phagocytes then transport them to lysosomes. KIM-1 is expressed in kidney (no other organs) of many species (human, rodents, monkeys, dogs, and zebrafish) and is shed from cells in vitro and in vivo after acute tubular necrosis. It has been reported that KIM-1 as a marker was more sensitive to different types of kidney insults (inflammation, nephrotoxicity, and cancer) than creatinine and urea. Thus, KIM-1 is a very useful indicator in the evaluation of kidney damage and the development of new drugs [57, 58]. In the current study, the high expression fold change of this protein after incubation of Vero cells with CCl_4_ indicates the induction of the renal cell injury, and this finding can be confirmed by the previous study of Huo et al. [58]. In addition, previous studies observed an elevation in KIM-1 gene expression and protein level which were in concomitant with the high ROS level and inflammation that mediated the acute renal injury [57].

The sharp decrease in the expression of KIM-1 after treatment with the studied extracts indicates restoration of the healthy state of Vero cells which is possibly owed to their phytochemical content (Fig. 2a). Hence, these ingredients induced an overall improvement of the CCl_4_-treated Vero cells by reducing the ROS level and in turn preventing the necrotic mediators, oxidative and inflammatory stress. Thus, the renal cell necrosis and injury were reversed followed by a decline in the expression of the KIM-1 protein.

## Conclusions

We have demonstrated that CCl_4_ was able to induce toxicity and necrosis in Vero cells by elevating oxidative stress and inflammation, which were previously proven to be implicated in chronic kidney diseases. Treatment with AVE, PCE, HVE, and CSE overwhelmed CCl_4_-induced toxicity by inhibiting main necrotic mediators, whereas AVE exhibited the most effective therapeutic potential in most of the studied parameters. In addition, the efficacy of AVE was higher to that of Ks drug; this may be owed to its important constituents, which possessed potent antioxidant and anti-inflammatory activities, all of which give AVE great therapeutic importance.

## Data Availability

The data that supported this article are available in Tables 1 and 2 and Figs. 1, 2, 3, 4, 5. The data sets analyzed during the present study are available from the corresponding author on the reasonable request.

## Abbreviations

*4-HCA*: 4-hydroxycinnamic acid
*ABTS*: 2,2-azino-bis (3-ethylbenzthiazoline-6-sulfonic acid
*AO*: Acridine orange
*AV*: *Ammi visnaga*
*BHT*: butylated hydroxytoluene
*Cs*: *Cymbopogon schoenanthus*
*Cy-3-glc*: cyanidin-3-glucoside
*DCFH-DA*: 2,7-Dichlorofluorescin diacetate
*DMEM*: Dulbecco’s Modified Eagle Medium
*DPPH*: α α-diphenyl-β-picrylhydrazyl
*EB*: Ethidium bromide
ED: Effective dose
*FBS*: Fetal bovine serum
*GPX*: Glutathione peroxidase
*GSH*: Reduced glutathione
HA: *Hordeum vulgare*
*KIM-1*: Kidney injury molecuole-1
Ks: Ketosteril
*MTT*: 3-(4,5-dimethylthiazol-2yl-)-2,5-diphenyl tetrazolium bromide
*NF-кB*: nuclear factor-kappa B
*PI*: Propidium iodide
PC: *Petroselinum crispum*
*QR*: quercetin
*RNS*: Reactive nitrogen species
*ROS*: Reactive oxygen species
*SOD*: Superoxide dismutase
*TAC*: Total antioxidant capacity
*TBA*: Thiobarbituric acid
*TBARS*: TBA reactive substances
*TMP*: Tetramethoxypropane
*UA*: Ursolic acid
